# Systematic Review and Meta-Analysis of Vortioxetine for the Treatment of Major Depressive Disorder in Adults

**DOI:** 10.3389/fpsyt.2022.922648

**Published:** 2022-06-24

**Authors:** Xinyan Zhang, Yuchun Cai, Xiaowen Hu, Christine Y. Lu, Xiaoyan Nie, Luwen Shi

**Affiliations:** ^1^Department of Pharmacy Administration and Clinical Pharmacy, School of Pharmaceutical Sciences, Peking University, Beijing, China; ^2^Department of Population Medicine, Harvard Medical School and Harvard Pilgrim Health Care Institute, Boston, MA, United States; ^3^International Research Center for Medicinal Administration, Peking University, Beijing, China

**Keywords:** vortioxetine, major depressive disorder, adults, randomized controlled trials, meta-analysis

## Abstract

**Objective:**

We aimed to compare the efficacy, acceptability, and tolerability of vortioxetine in the treatment of Major Depressive Disorder (MDD) in adults.

**Method:**

We searched PubMed, Embase, Web of Science, Cochrane Central Register of Controlled Clinical Trials (CENTRAL), and www.ClinicalTrials.gov for randomized controlled trials that examined vortioxetine vs. placebo or other antidepressants for the treatment of MDD from database inception to August 30, 2021, using keywords Vortioxetine, Brintellix, Trintellix, LuAA21004, major depressive disorder, mood disorder, affective disorder, and MDD. We identified 789 publications after removing duplicates. After screening, 20 eligible randomized controlled trials were identified, of which 19 were included in the final meta-analysis. We included adults (aged 18 years and older) with a primary diagnosis of MDD. Two review authors independently selected the studies and extracted data. We extracted data on study characteristics, participant characteristics, intervention details and outcome measures in terms of efficacy, acceptability, and tolerability. Analyses were performed using random-effects models, and outcomes were pooled as risk ratios (RRs) and standardized mean differences (SMDs).

**Results:**

In total, 20 studies (8,547 participants) met the inclusion criteria. Vortioxetine outperformed the placebo in efficacy outcomes, including response (RR 1.35, 95% CI 1.23–1.48; *P* < 0.001), remission (RR 1.33, 95% CI 1.17–1.52; *P* < 0.001), and cognitive function (SMD 0.34, 95% CI 0.16–0.52; *P* = 0.0003). Compared with the serotonin noradrenaline reuptake inhibitors (SNRIs), vortioxetine had better tolerability (RR 0.90, 95% CI 0.86–0.94; *P* < 0.001) but no significant difference in response (RR 0.91, 95%CI 0.82–1.00; *P* = 0.06) or remission (RR: 0.99, 95% CI 0.81–1.20, *P* = 0.88). Vortioxetine had no difference in response (RR 1.08, 95% CI 0.88–1.32; *P* = 0.46), remission (RR 1.00, 95% CI 0.41–2.44; *P* = 1.00) comparing with selective serotonin reuptake inhibitors (SSRIs).

**Conclusions:**

Vortioxetine is more advantageous over placebo in treating MDD among adults, but no significant difference compared to SNRIs and SSRIs in general.

**Systematic Review Registration:**

https://www.crd.york.ac.uk/PROSPERO/display_record.php?ID=CRD42021278355, identifier: CRD42021278355.

## Introduction

Major depressive disorder (MDD) is characterized by depressed mood, loss of pleasure, low energy, and feelings of low self-worth (1). According to the World Health Organization, MDD is the most prevalent psychiatric disorder globally (2), affecting more than 350 million individuals worldwide (3). MDD reduced the patients' quality of life (4), lowered social productivity (5), increased their suicide attempts (6), and burdened their financial status (7), resulting in significant and far-reaching adverse medical and social consequences.

Various pharmacological and psychological interventions have been used to treat MDD. Major pharmacotherapies include selective serotonin reuptake inhibitors (SSRIs), serotonin noradrenaline reuptake inhibitors (SNRIs), conventional tricyclic antidepressants (TCAs), monoamine oxidase inhibitors (MAOIs), and other antidepressant agents. Most guidelines by far recommend SSRIs as the first-line treatment due to their minor adverse effects (8, 9). Though antidepressants approved for treating MDD is increasing, only 50% of patients have sufficient response to treatment with adequate duration (10), about 40% of patients who achieved remission are at risk of relapse within a year (11, 12). Therefore, discovering antidepressants with superior effectiveness and tolerability is in urgent clinical need in treating MDD (13).

Vortioxetine is a novel antidepressant with multimodal activities that was first licensed by the United States' Food and Drug Administration (FDA) to treat MDD in adults in 2013 (14–16), and has a distinct pharmacological profile from current treatment options by both directly modulating 5-HT receptors and inhibiting the serotonin transporter (14, 15). The clinical trials demonstrated significantly improved Montgomery-Åsberg Depression Scale (MADRS) scores and increased in response rate in those with depression who were administered vortioxetine compared with placebo (5, 17–19), and have also shown a favorable tolerability profile and improved cognitive dysfunction (20). With a growing number of studies focusing on the vortioxetine treatment of MDD in recent years, this study aimed to conduct a systematic review to assemble the efficacy, acceptability, and tolerability of vortioxetine in treating MDD compared to placebo, SNRIs and SSRIs.

## Method

### Search Strategy and Selection Criteria

For this systematic review and meta-analysis, we searched PubMed, Embase, Web of Science, and the Cochrane Central Register of Controlled Clinical Trials (CENTRAL) for studies published from database inception to August 30, 2021. To identify ongoing or unpublished studies, we searched ClinicalTrials.gov, using keywords “Vortioxetine,” “Brintellix,” “Trintellix,” “LuAA21004,” “major depressive disorder,” “mood disorder,” “affective disorder,” and “MDD.” Manual searches were conducted in the reference sections of the published meta-analyses and reviews to identify potentially relevant articles. We imposed no language, publication status, or publication type restrictions.

### Protocol and Registration

This study was registered on PROSPERO (CRD42021278355) and conducted following the Preferred Reporting Items for Systematic Reviews and Meta-Analyses recommendations (21).

### Types of Studies

Only RCTs were included in this review.

### Types of Participants

We included adults (aged 18 years and older) with a primary diagnosis of MDD according to the standard operationalized diagnostic criteria.

### Types of Interventions

Studies with vortioxetine as monotherapy vs. any comparator intervention were included. Identified comparisons include placebo and other antidepressants, such as SSRIs, SNRIs, TCAs, MAOIs, and other antidepressant agents. We combined various dosages of the same comparator into a single group.

### Types of Outcome Measures

The efficacy outcome includes response (≥50% reduction on an observer-rated depression scale), remission [MADRS ≤ 10, Hamilton Depression Rating Scale (HAM-D) total score ≤ 7], tolerability (total number of participants experiencing at least one adverse event), acceptability (dropout due to adverse events) and cognitive function. For response or remission rates, we gave preference to MADRS outcomes over HAM-D. Other validated depression scales are also acceptable if neither was reported (8).

### Study Selection

Two independent reviewers screened the titles and abstracts using the systematic review program, obtained relevant studies in full, and determined separately whether the study met the inclusion criteria (with an average kappa of 0.89). Any disagreements between reviewers were resolved *via* discussion to achieve consensus, and a third reviewer consulted if the two initial reviewers could not reach a consensus.

### Data Extraction

Two reviewers' extracted data into a pre-piloted, standardized data extraction form in Microsoft Excel 2019, detailing the sample size, mean age, diagnostic criteria, interventions, comparisons, the dose of administration, and outcomes of each study. We contacted the authors for additional information when data were not reported in full. Data were extracted as a single study when we identified multiple associated publications. For randomized controlled trials, we assessed the risk of bias using the Cochrane Risk of Bias Tool to generate allocation sequence, allocation concealment, masking of study personnel and participants, masking of outcome assessor, attrition bias, and selective outcome reporting bias. According to the criteria set out in the Cochrane Handbook for Systematic Reviews of Interventions (22), each trial was classified into low, unclear, or high risk. At least two independent reviewers selected the studies and assessed the risk of bias.

### Statistical Analysis

We used Review Manager (version 5.3; Cochrane Collaboration, Oxford, United Kingdom) and the STATA version 15 for all analysis. Continuous outcomes were pooled as standardized mean differences (SMDs). Dichotomous data were pooled as risk ratios (RRs). We contacted the authors for additional data when the reported information was insufficient to calculate the mean and the standard deviation. Heterogeneity between studies was assessed using *I*^2^ values. Thresholds for the interpretation of heterogeneity were consistent with those of the Cochrane Collaboration [*I*^2^ values of 0%−40% might not be important; 30%−60% may represent moderate heterogeneity; 50%−90% may represent substantial heterogeneity; and 75%−100% had high levels of heterogeneity; Higgins and Green, (23)]. A fixed-effects model was used if heterogeneity was moderate or less; otherwise, a random-effects model was applied. For all analysis, statistical significance was set at *P* < 0.05. We performed sensitivity analysis by excluding studies judged at high risk of bias.

### Evaluation of Publication Bias

Publication bias was assessed by visually inspecting funnel plots and Egger's linear regression intercept in Stata [Egger et al. (24)] in our meta-analysis. Significant publication bias was defined as a *P*-value <0.1.

## Results

### Study Selection

**Figure 1:**
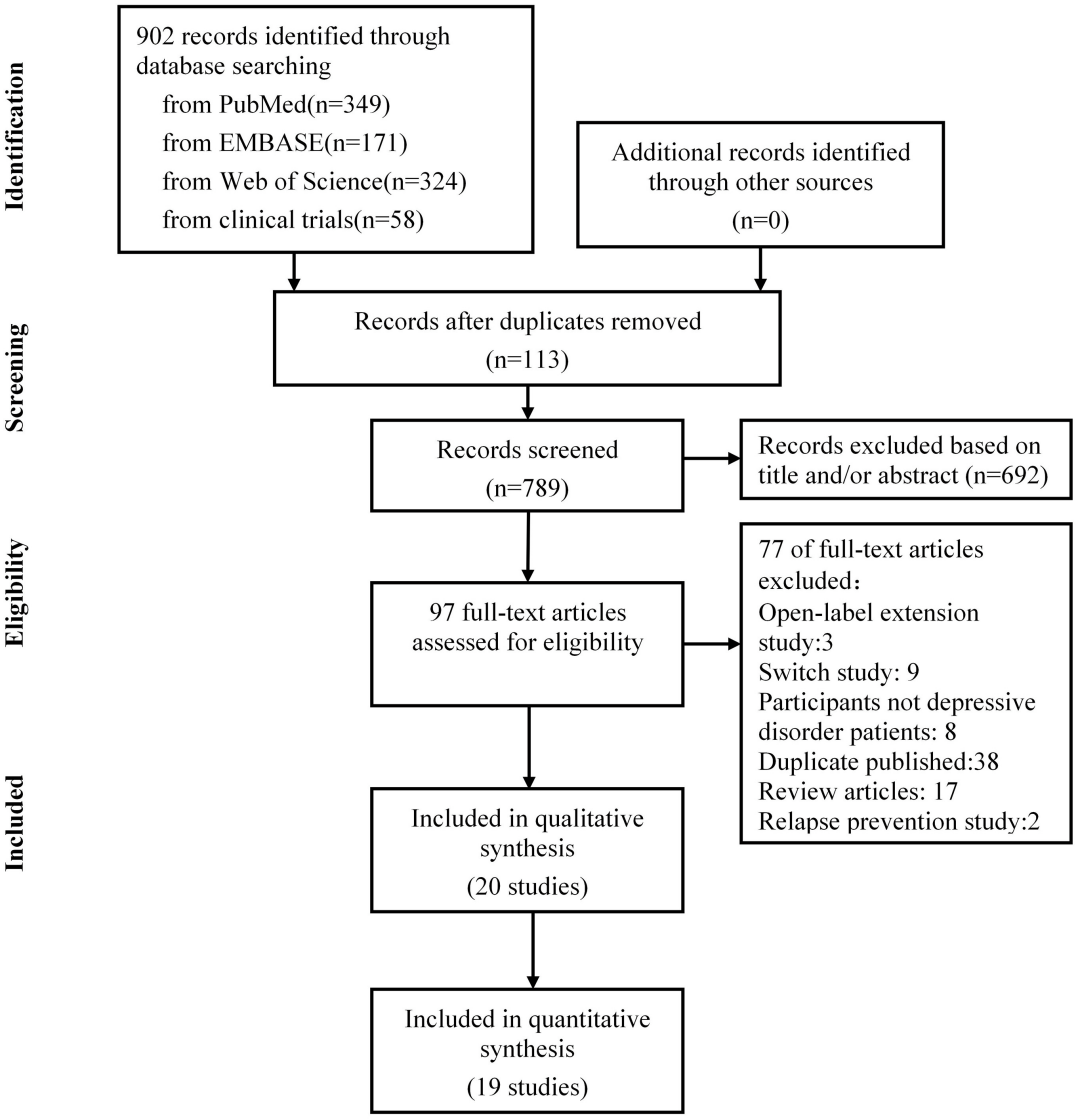
Flowchart of the selection of the clinical trials.

The literature search was updated on September 2021. Nine hundred and two publications were identified through electronic search, manual search, author contact, and trial registries. We retrieved 97 full-text articles for detailed examination, excluded 77 articles that did not meet the eligibility criteria, and removed 38 publications reporting the same trials. Further, we excluded eight studies that did not meet our inclusion criteria for depressive disorder patients, two studies of relapse prevention, 17 reported review articles, three open-label extension studies, and nine trials with inadequate response. After screening, 20 eligible randomized controlled trials were identified, of which 19 were included in the final meta-analysis. The PRISMA flowchart is presented in Figure 1.

### Description of Studies

Table 1 summarized the characteristics of each included randomized controlled trial. Overall, this study comprised 8,547 participants, of which 4,598 were allocated to vortioxetine, 2,538 to the placebo, 98 to SSRIs, 1,313 to SNRIs. Among the included trials, eight were triple-armed with vortioxetine, an active comparison, and a placebo; 12 were double-armed, including nine placebo-controlled and three active comparisons trials. Nine trials compared vortioxetine with placebo only; two compared the efficacy of vortioxetine with that of SSRIs; eight compared vortioxetine with SNRIs. The vortioxetine dosage ranged from 5 to 20 mg/day in the studies. Three studies lasted 6 weeks, and 17 studies lasted 8 weeks.

**Table 1:** Characteristics of included studies.

**Study name**	**Interventions**	**Control**	**No. randomized**	**Mean age (SD)**	**Sex**	**Dosing schedule**	**Length of trial (weeks)**	**Diagnostic criteria**	**Recruitment**	**Primary endpoint assessment**
Alvarez (25)	Vortioxetine 5 mg Vortioxetine 10 mg Venlafaxine 225 mg	Placebo	IG = 105/109/101 CG = 114	IG = 42 (10.9)/43.8 (11.6)/42.3 (13.1) CG = 45 (10.3)	63% women	Fixed	6	DSM-IV	Cross-Continental	MADRS
Baldwin (26)	Vortioxetine 5 mg Vortioxetine 10 mg Duloxetine 60 mg	Placebo	IG = 152/159/153 CG = 157	IG = 43.3 (12.5)/44.7 (13.1)/45.2 (13.1) CG = 45.3 (12)	68% women	Fixed	8	DSM-IV	Cross-Continental	MADRS
Boulenger et al. (5)	Vortioxetine 15 mg Vortioxetine 20 mg Duloxetine 60 mg	Placebo	IG = 158/152/151 CG = 147	IG = 48.1 (13.1)/47 (14.6)/46.2 (13.4) CG = 45.6 (13.6)	66% women	Fixed	8	DSM-IV	Cross-Continental	MADRS
Henigsberg et al. (15)	Vortioxetine 5 mg Vortioxetine 10 mg	Placebo	IG = 140/140 CG = 140	IG = 46.4 (12.3)/47.3 (11.9) CG = 46.4 (12.3)	61% women.	Fixed	8	DSM-IV	Cross-Continental	HAMD-24
Jacobsen et al. (18)	Vortioxetine 10 mg Vortioxetine 20 mg	Placebo	IG = 157/155 CG = 150	IG = 42.3 (11.6)/43.1 (12.0) CG = 43.1 (13.1)	73% women	Fixed	8	DSM-IV	North America	MADRS
Jain (27)	Vortioxetine 5 mg	Placebo	IG = 300 CG = 300	IG = 42.4 (12.7) CG = 42.5 (13.0)	58% women	Fixed	6	DSM-IV	North America	HAMD-24
Katona (28)	Vortioxetine 5 mg Duloxetine 60 mg	Placebo	IG = 145/156 CG = 151	IG = 70.3 (4.4)/70.5 (4.8) CG = 70.9 (5.5)	66% women	Fixed	8	DSM-IV	Cross-Continental	HAMD-24
Mahableshwarkar (29)	Vortioxetine 5 mg Duloxetine 60 mg	Placebo	IG = 153/153 CG = 152	IG = 42.6 (13.7)/43.1 (13.9) CG = 42.7 (14.4)	63% women	Fixed	8	DSM-IV	North America	HAMD-24
Mahableshwarkar (17)	Vortioxetine 15 mg Vortioxetine 20 mg Duloxetine 60 mg	Placebo	IG = 161/147/154 CG = 152	IG = 42.4 (12.6)/43.1 (12.3)/42.8 (12.4) CG = 43.4 (12.2)	74% women	Fixed	8	DSM-IV	North America	MADRS
Mahableshwarkar (30)	Vortioxetine 10 mg Vortioxetine 15 mg	Placebo	IG = 160/157 CG = 152	IG = 46.2 (11.8)/45.2 (11.9) CG = 43.8 (13.5)	65% women	Fixed	8	DSM-IV	North America	DSST
Mahableshwarkar (31)	Vortioxetine 10–20 mg Duloxetine 60 mg	Placebo	IG = 194/198 CG = 210	IG = 45 (12.1)/44.2 (12.2) CG = 45.7 (11.5)	70% women	Fixed Flexible Fixed	8	DSM-IV	Cross-Continental	MADRS
McIntyre et al. (19)	Vortioxetine 10 mg Vortioxetine 20 mg	Placebo	IG = 198/197 CG = 207	IG = 45.6 (12.1)/45.4 (12.2) CG = 46.1 (11.8)	66% women	Fixed	8	DSM-IV	Cross-Continental	DSST and RAVLT
Takeda (32)	Vortioxetine 5 mg Vortioxetine 10 mg	Placebo	IG = 124/119 CG = 123	IG = 37.6 (10.7)/38.8 (10.8) CG = 38.8 (11)	47% women	Fixed	8	DSM-IV	Asia	MADRS
Wang (33)	Vortioxetine 10 mg	Venlafaxine 150 mg	IG = 213 CG = 230	IG = 39.6 (12.4) CG = 40.7 (12.3)	60% women	Fixed	8	DSM-IV	Asia	MADRS
Liebowitz (34)	Vortioxetine 10 - 20 mg	placebo	IG = 21 CG = 21	IG = 40.8 (14.5) CG = 42.2 (12.6)	60% women	Flexible	8	DSM-5	North America	CGI
Inoue et al. (35)	Vortioxetine 10 mg Vortioxetine 20 mg	placebo	IG = 164/165 CG = 164	IG = 39.5 (10.5)/40.0 (10.6) CG = 40.4 (11.3)	45% women	Fixed	8	DSM-IV	Japan	MADRS
Nishimura (36)	Vortioxetine 5 mg Vortioxetine 10 mg Vortioxetine 20 mg	Placebo	IG = 152/144/150 CG = 154	IG = 43.6 (11.57)/44.2 (11.89)/45.7 (10.9) CG = 44 (11.79)	63% women	Fixed	8	DSM-IV	Cross-Continental	MADRS
Borhannejad et al. (37)	Vortioxetine 15 mg	Sertraline 75 mg	IG = 30 CG = 30	IG = 71.84 (7.75) CG = 69.44 (8.71)	74% women	Fixed	6	DSM-5	Iran	HAM-D-17
Baune et al. (38)	Vortioxetine 10 mg Paroxetine 20 mg	Placebo	IG = 48/48 CG = 54	IG = 45.0 (12.7)/47.3 (12.0) CG = 46.3 (11.5)	66% women	Fixed	8	DSM-IV	Cross-Continental	DSST
Levada et al. (39)	Vortioxetine 10–20 mg	Escitalopram 10–20 mg	IG = 36 CG = 20	IG = 37.3 (11.0) CG = 37.2 (12.4)	59% women	Fixed	8	DSM-IV	Ukraine	MADRS

*CG, control group; IG, intervention group; DSST, Digit Symbol Substitution Test; CGI-S, Clinical Global Impression–Severity; HAM-D, Hamilton Depression Rating Scale; RAVLT, Rey Auditory Verbal Learning Test; MADRS, Montgomery-Åsberg Depression Scale; DSM, Diagnostic and Statistical Manual of Mental Disorders*.

For the study outcomes, 19 studies provided the response and remission rates. All but two studies reported tolerability and acceptability. Seventeen studies reported dropouts owing to adverse effects. Eleven studies used MADRS to measure the outcome, four used the HAM-D-24, three used the Digit Symbol Substitution Test (DSST), others used the Clinical Global Impression–Severity (CGI-S), the HAM-D-17, and the Rey Auditory Verbal Learning Test (RAVLT) as the outcome measure.

**Figure 2:**
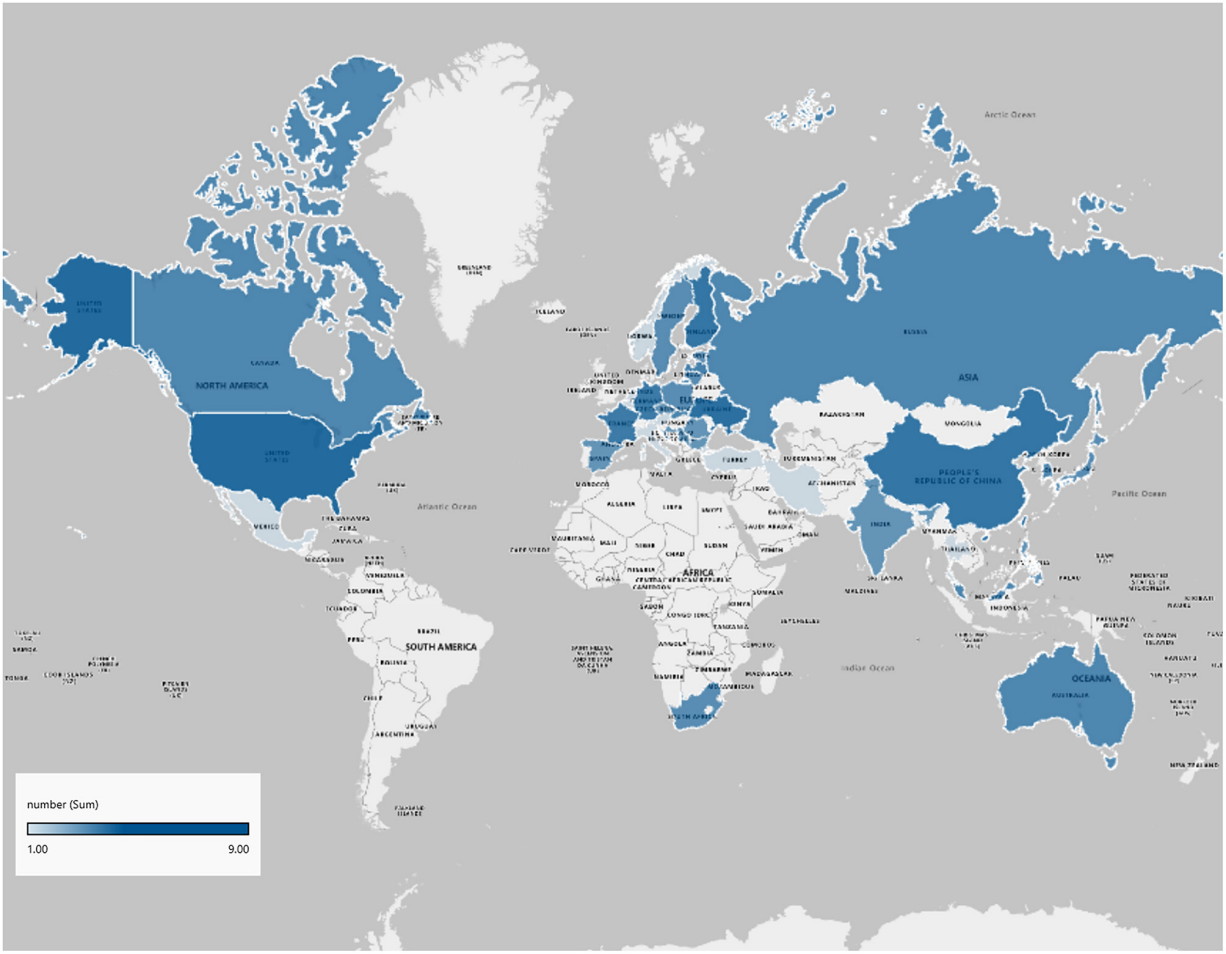
Countries participating in trials.

Regarding the representativeness of participants, nine studies were multinational across continents and nine were multicenter studies conducting in a single nation in our analysis and two (10%) were conducted at a single center. Figure 2 shows an overview of the countries where participants were recruited.

**Figure 3:**
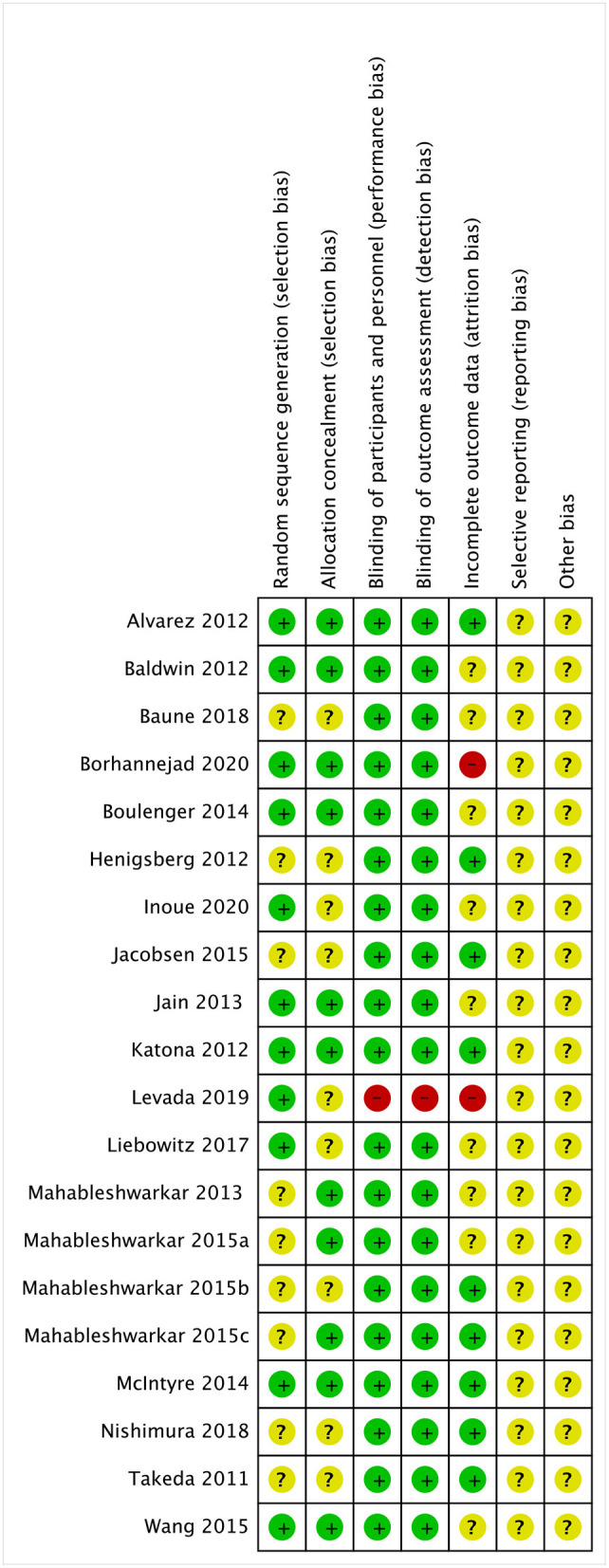
Risk of bias summary.

Graphical representations of the risk of bias assessment are shown in Figure 3. Briefly, nine studies (45%) of the randomized controlled trials did not report adequate randomization sequence generation and concealment, while the 19 studies (95%) were at low risk of bias for masking of participants, personnel, and outcome assessors.

### Effects of Interventions

We presented the meta-analysis results by grouping the comparators into three categories: placebo, SNRIs, and SSRIs.

#### Vortioxetine vs. Placebo

Sixteen studies with 6755 participants provided data comparing vortioxetine vs. placebo.

##### Response

**Figure 4:**
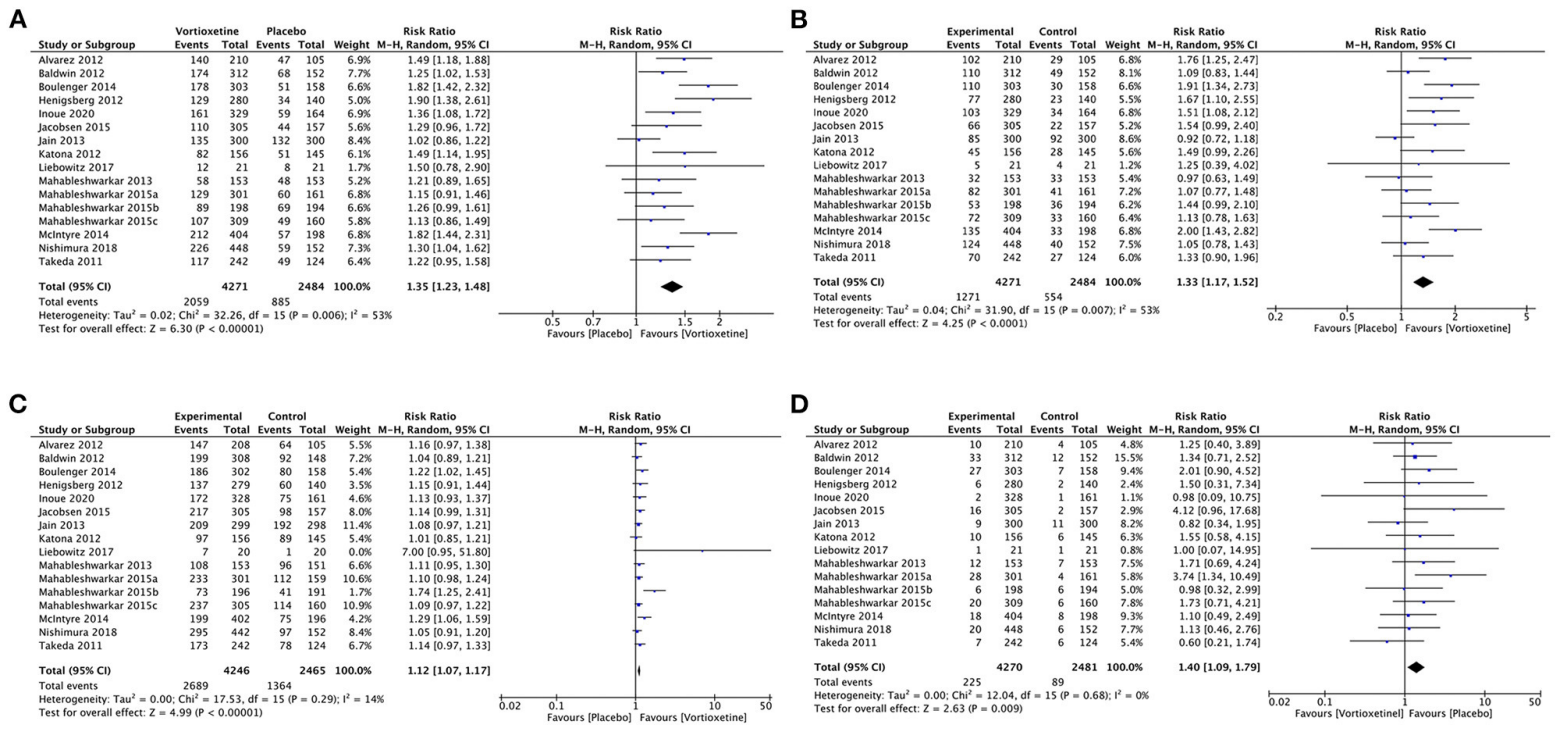
Forest plot of comparison on vortioxetine vs. placebo **(A)** Response, **(B)** remission, **(C)** tolerability, and **(D)** acceptability.

Vortioxetine had a significantly higher response rate than placebo (RR 1.35, 95% CI = 1.23–1.48; *P* < 0.001; *I*^2^ = 53%; Figure 4A).

##### Remission

Vortioxetine was associated with a higher remission rate than the placebo (RR 1.33, 95% CI = 1.17–1.52; *P* < 0.001; *I*^2^ = 53%; Figure 4B).

##### Tolerability

Patients using vortioxetine experienced more adverse effects than the placebo (RR 1.12, 95% CI = 1.07–1.17; *P* < 0.001; *I*^2^ = 14%; Figure 4C).

##### Acceptability

Vortioxetine was associated with a higher dropout rate due to adverse events than the placebo (RR 1.40, 95% CI = 1.09–1.79; *P* = 0.009; *I*^2^ = 0%; Figure 4D).

##### Cognitive Function

**Figure 5:**
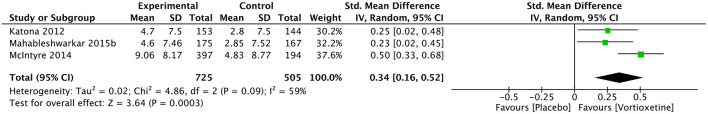
Forest plot of comparison on cognitive function on vortioxetine vs. placebo.

Vortioxetine significantly improved DSST score than the placebo (SMD 0.34, 95% CI = 0.16–0.52; *P* = 0.0003; *I*^2^ = 59%; Figure 5).

#### Vortioxetine vs. SNRIs

Eight studies including 3,159 participants provided data comparing vortioxetine to SNRIs.

##### Response

**Figure 6:**
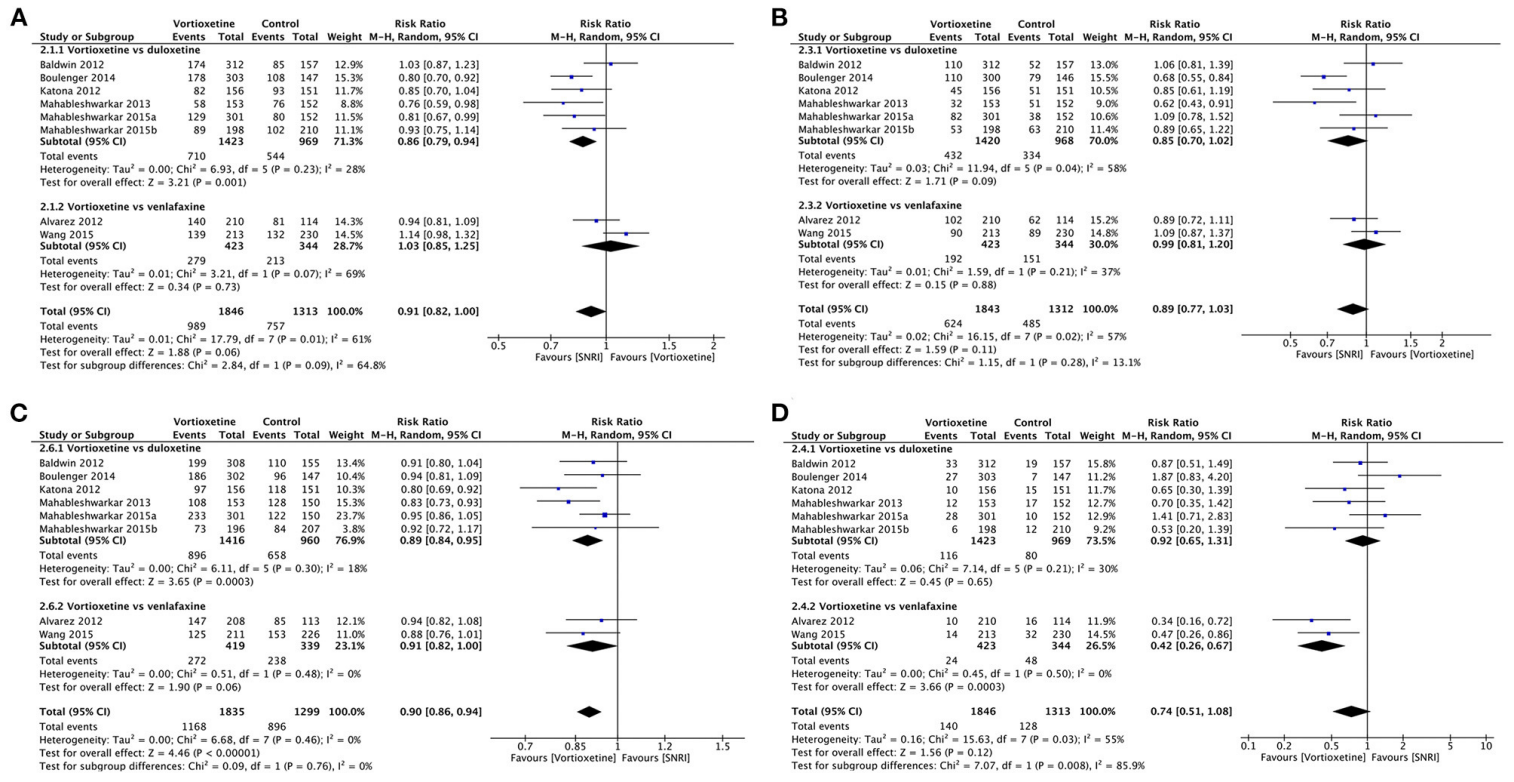
Forest plot of comparison on vortioxetine vs. SNRIs **(A)** Response, **(B)** remission, **(C)** tolerability, and **(D)** acceptability.

Vortioxetine had no significant difference in response rate compared with SNRIs in general (RR 0.91, 95% CI = 0.82–1.00; *P* = 0.06; *I*^2^ = 61%). Specifically, as shown in Figure 6A, based on the results of six studies (*n* = 2,392), the response rates were significantly lower for vortioxetine than duloxetine; the pooled RR was 0.86, (95% CI = 0.79–0.94; *P* = 0.001; *I*^2^ = 28%), while two studies (*n* = 767) showed that there was no difference in response rates compared to venlafaxine (RR 1.03, 95% CI = 0.85–1.25; *P* = 0.73; *I*^2^ = 69%; Figure 6A).

##### Remission

There was no significant difference in the remission rates between vortioxetine and venlafaxine (RR: 0.99, 95% CI = 0.81–1.20, *P* = 0.88; *I*^2^ = 37%), vortioxetine and duloxetine (RR 0.85, 95% CI = 0.70–1.02; *P* = 0.09; *I*^2^ = 58%), or vortioxetine and SNRIs in general (RR 0.89, 95% CI = 0.77–1.03; *P* = 0.11; *I*^2^ = 57%; Figure 6B).

##### Tolerability

Participants treated with vortioxetine experienced fewer adverse effects than those treated with SNRIs (RR 0.90, 95% CI = 0.86–0.94; *P* < 0.001; *I*^2^ = 0%). In subgroup analyses, vortioxetine treatment had fewer adverse effects than duloxetine (RR 0.89, 95% CI = 0.84–0.95; *P* < 0.001; *I*^2^ = 18%), but no significant difference is observed between vortioxetine and venlafaxine (RR 0.91, 95% CI = 0.82–1.00; *P* = 0.06; *I*^2^ = 0%; Figure 6C).

##### Acceptability

Vortioxetine had no significant difference in dropout rate due to adverse events comparing with SNRIs in general (RR 0.74, 95% CI = 0.51–1.08; *P* = 0.12, *I*^2^ = 55%). Specifically, there was no statistically significant difference between vortioxetine and duloxetine in total dropout rates due to adverse events (RR 0.92, 95% CI = 0.65–1.31; *P* = 0.65; *I*^2^ = 30%), but vortioxetine is superior when compared with venlafaxine (RR 0.42, 95% CI = 0.26–0.67; *P* < 0.001; *I*^2^ = 0%; Figure 6D).

#### Vortioxetine vs. SSRIs

Two studies included 126 participants that provided efficacy and acceptability data comparing vortioxetine and SSRIs.

**Figure 7:**
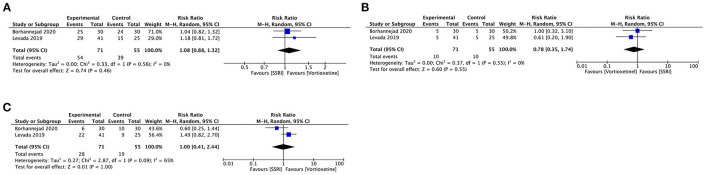
Forest plot of comparison on vortioxetine vs. SSRIs **(A)** Response, **(B)** remission, and **(C)** acceptability.

Vortioxetine had no difference in response rate (RR 1.08, 95% CI = 0.88–1.32; *P* = 0.46; *I*^2^ = 0%), remission rate (RR 1.00, 95% CI = 0.41–2.44; *P* = 1.00; *I*^2^ = 65%) or acceptability (RR 0.78, 95% CI = 0.35–1.74; *P* = 0.55; *I*^2^ = 0%) comparing with SSRIs (Figure 7).

### Sensitivity Analysis

Effect estimates were consistent with overall summary effect estimates for outcomes when contributing data excluded low quality studies.

### Publication Bias

**Figure 8:**
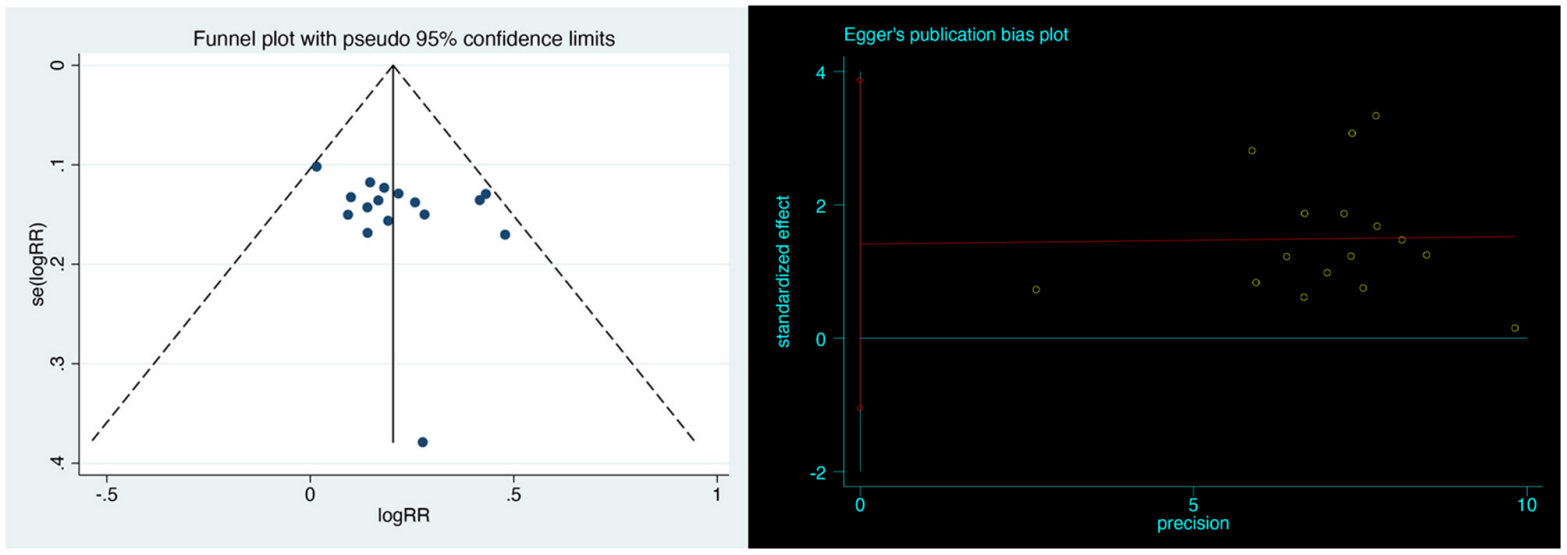
Publication bias for vortioxetine in all included studies.

Visual inspection of funnel plots revealed no obvious publication bias. Egger's linear regression test further justified that the publication bias was non-significant (*P* = 0.239; Figure 8).

## Discussion

This comprehensive meta-analysis focused on the efficacy, acceptability, tolerability and cognitive function of vortioxetine as monotherapy among adult patients with MDD. We found significant advantages in the efficacy, tolerability and cognitive function of vortioxetine compared to the placebo, aligning with the existing literature. However, vortioxetine has no significant advantage in efficacy compared with SNRIs and SSRIs, but it dominates SNRIs in terms of tolerability.

Vortioxetine has been approved in more than 80 countries worldwide, with efficacy supported by numerous randomized controlled trials (35, 40, 41). Similar to previous studies, we found that vortioxetine treatment significantly improved patients' chances of response and remission rates. However, the vortioxetine in individual trials showed a nonconsistency benefit of treatment vs. placebo. It is noteworthy that 20 mg/day of vortioxetine did not outperform placebo in the United States. Possible reasons for difference may be related to disparities in ethnicity, geographic location, and the ability to detect efficacy in clinical trials (42).

Impairment in cognitive function is increasingly recognized as a core deficit in patients with MDD. Some residual symptoms, including impairments in executive function and attention often persist independently even after remission of depressive symptoms (43–45). Our evidence supports that vortioxetine has a statistically significant effect on the cognitive functions in comparison with the placebo. Vortioxetine is the only FDA-approved pharmacological agent for the treatment of MDD that specifically targets cognitive dysfunction (46), and increasing evidence has demonstrated the clinical benefits of vortioxetine on cognitive function. Vortioxetine was statistically more efficacious on cognitive function than escitalopram, nortriptyline, SSRIs and TCAs (38) and their impact independent of depressive symptoms (47), and the superiority compared with the placebo in speed of processing, verbal learning, and memory (17). Further research is needed to understand the relationship between the differential effects on cognition and unique pharmacological profile.

In this study, there was no difference in terms of efficacy between vortioxetine and SNRIs, and the results of the subgroup analysis showed that the response rate of vortioxetine might be lower than that of duloxetine and no different from venlafaxine, which is inconsistent with the results from previous meta-analysis which show the superior efficacy of venlafaxine over duloxetine (48). One explanation for this is that the pooled result might be biased because it is based only on a small number of studies. However, We also revealed that vortioxetine may have advantages over SNRIs in terms of tolerability profile, which was confirmed by the results reported in other head-to-head studies (20, 49, 50), and vortioxetine has generally been associated with a lower prevalence of adverse events compared with duloxetine and extended-release venlafaxine. Tolerability has been used as an indirect index of drug effectiveness (51); therefore, the better tolerability profile of vortioxetine may increase the probability of patient adherence and remission.

In the comparison with SSRIs, we found no significant differences in the efficacy and acceptability of vortioxetine. SSRIs are usually recommended as first-line treatment for MDD. Therefore, direct comparisons of these antidepressants may help to better guide the clinical application of vortioxetine. However, the current quantitative results are based only on two clinical trials with small sample sizes. A randomized trial of older patients with MDD showed no significant differences in the efficacy or safety of vortioxetine vs. sertraline at week 6 of the trial (37). However, compared to escitalopram, vortioxetine treatment improved overall cognitive performance, which led to higher response and remission rates (39). The evidence on the effect of SSRIs may need to be evaluated in additional high-quality clinical trials.

Several published systematic reviews and meta-analyses yielded evidence of efficacy for vortioxetine in depression. One narrative review gave an overview of the vortioxetine studies and reported results from 10 RCTs in adults with depression without pooling the results (49). Two other meta-analyses focused on specific doses of vortioxetine 5 mg or 10 mg compared to placebo (52, 53). Koesters et al. (54) conducted a comprehensive review of the effects of vortioxetine in the treatment of MDD in adults, but included RCTs only up to 2016. The present work is the most comprehensive published to date focusing on the efficacy of vortioxetine as monotherapy for MDD. Our study, however, is subject to several limitations. First, differences in the patient baseline characteristics, disease severity, and dose of vortioxetine among the identified clinical trials may cause the high heterogeneity of the results. Second, due to the limited availability of original trials, our pooled results might not provide sufficient evidence in comparison to other antidepressants in terms of cognitive function, as well as also not allowing for corresponding subgroups analysis. Third, our evidence applying the DSST as a single cognitive test comparison means that the comparison and pooling of data was limited in the analysis. Therefore, future research could improve the standardization of cognitive testing by combining self-reports and objective cognitive tests. Finally, most of the clinical trials included in this meta-analysis were funded by pharmaceutical companies, so the underlying conflicts of interest may influence the results.

## Conclusions

In conclusion, our findings indicated that vortioxetine for the treatment of MDD in adults has significant advantages over placebo in terms of efficacy, tolerability, and cognitive function. In comparison to SNRIs and SSRIs in terms of efficacy, there was no statistically significant difference for vortioxetine, which may be similarly effective but has advantages over the SNRIs in terms of tolerability. Further studies including head-to-head comparisons with SNRIs and SSRIs are needed to supplement and verify the efficacy to define the role of vortioxetine in the treatment of depression.

## Data Availability Statement

The original contributions presented in the study are included in the article/supplementary material, further inquiries can be directed to the corresponding author.

## Author Contributions

XZ designed the study and registered on PROSPERO, searched the online databases, assessed the risk of bias of included studies, carried out the initial statistical analysis, drafted the initial manuscript, and revised the manuscript. CL searched the online databases, assessed the risk of bias of included studies, reviewed, and revised the manuscript. XH searched the online databases, evaluated all searched citations, and extracted the primary data. YC searched the online databases, evaluated all searched citations, analyzed the data, and extracted the data. XN and LS designed this study, reviewed, and revised the manuscript. All authors approved the final version of the manuscript.

## Conflict of Interest

The authors declare that the research was conducted in the absence of any commercial or financial relationships that could be construed as a potential conflict of interest.

## Publisher's Note

All claims expressed in this article are solely those of the authors and do not necessarily represent those of their affiliated organizations, or those of the publisher, the editors and the reviewers. Any product that may be evaluated in this article, or claim that may be made by its manufacturer, is not guaranteed or endorsed by the publisher.
